# An Electronic Nose Based on Coated Piezoelectric Quartz Crystals to Certify Ewes’ Cheese and to Discriminate between Cheese Varieties

**DOI:** 10.3390/s120201422

**Published:** 2012-02-01

**Authors:** Vânia F. Pais, João A. B. P. Oliveira, Maria Teresa S. R. Gomes

**Affiliations:** CESAM & Department of Chemistry, University of Aveiro, Aveiro 3810-193, Portugal; E-Mails: vaniapais@hotmail.com (V.F.P.); jabpo@ua.pt (J.A.B.P.O.)

**Keywords:** electronic nose, piezoelectric quartz crystal, cheese discrimination, ewe’s cheese

## Abstract

An electronic nose based on coated piezoelectric quartz crystals was used to distinguish cheese made from ewes’ milk, and to distinguish cheese varieties. Two sensors coated with Nafion and Carbowax could certify half the ewes’ cheese samples, exclude 32 cheeses made from cow’s milk and to classify half of the ewes’ cheese samples as possibly authentic. Two other sensors, coated with polyvinylpyrrolidone and triethanolamine clearly distinguished between Flamengo, Brie, Gruyère and Mozzarella cheeses. Brie cheeses were further separated according to their origin, and Mozzarella grated cheese also appeared clearly separated from non-grated Mozzarella.

## 1. Introduction

Food product authenticity is of great concern both for consumers and manufacturers. Fraud, namely where products hold a certificate of origin, must be detected. There is a need for low cost, portable devices that would allow inspectors to use them in non-laboratory environments, to avoid the delay of sending samples for analysis. In the absence of such a device, an inexpensive technique that does not require dedicated facilities would also be of great help.

Piezoelectric quartz crystals are inexpensive, as they are standard electronic components, and show a remarkable sensitivity to mass. When coated with a compound that interacts with an analyte, forming a product with different mass, they can be used as chemical sensors. An array of crystals with different coatings can be comparable to a human nose with different receptors. The cost of such a device increases as the frequency of several crystals must be read simultaneously, but even so, they are much cheaper than a gas chromatograph.

The application of electronic noses to dairy products is not new [1], but Ampuero *et al.* [1] have referred to the difficulties found due to matrix complexity. In fact, most of the work has been carried out by GC/MS, also called an electronic nose in some reports [2–4], although no similarity with a human nose can be established. Mass spectrometry without prior chromatographic separation has also been used for dairy products [5–8]. These bulky and expensive instruments allowed the identification of aroma compounds, only possible with electronic noses if analytes are limited in number and after extensive training [9]. Another possibility consists of the evaluation of dairy products by a sensory panel, which is a subjective method, very dependent on the expertise of the panel members and influenced by human limitations.

Until now, electronic noses used in cheese evaluation have been either commercial [10–13] or self-assembled in the laboratory [14,15], and have used sensors based on two different types of transducers: metal oxide semiconductors (MOS) [10–14], or bulk acoustic wave sensors (BAW) [15]. Data processing from all sensors was mandatory, and usually no attempt was made to reduce the number of sensors, or to identify those that were really needed, even when a single target compound was defined [15]. In this work we have tried to limit the number of sensors, and to make the discrimination of cheese by milk type or variety as simple as possible. Sensors have been limited to two for each problem. This limitation on the number of sensors made the analysis simpler and users do not need to have any chemometric know-how.

The volatile compounds in the cheese samples were extracted by the static headspace method. Solid phase microextraction (SPME) allowed many volatile compounds to be collected without the use of any solvent.

Cheeses selected for e-nose analysis included some of the world’s best known varieties including soft cheeses, such as Camembert and Brie, fresh cheeses, semi-hard and hard cheeses, such as Gruyère, Grana Padano, Gouda, and Manchego, and many others. Cheeses of the same variety produced in different regions were also included in the sample set.

This work showed that an electronic nose with just four sensors could be used as a first screening method to distinguish between cheese made from ewe’s milk and cheese produced from cows’, or mixed milk (two sensors required), as well as several varieties of cheeses, such as Flamengo, Brie, Gruyère, and Mozzarella (two other sensors needed). The Brie cheeses from different origins were also distinguished, and, among the Mozzarellas, grated cheese and goat’s cheese were also separated.

## 2. Experimental Section

### 2.1. Chemicals and Samples

All sensors used 9 MHz piezoelectric quartz crystals. Sensor 1 was coated with 1,10-decanedithiol (TCI D0015), sensor 2 with Nafion 117 solution (Fluka 70160), sensor 3 with Carbowax (Supelco 21032), sensor 4 with Tenax TA (Supelco 21009-U), sensor 5 with poly(dimethylsiloxane) (ABCR 76189), sensor 6 with manganese(II) phthalocyanine (Aldrich 379557), sensor 7 with poly(vinyl alcohol) (Fluka 81381), sensor 8 with polyvinylpyrrolidone (Fluka 81420), sensor 9 with 6-mercapto-1-hexanol (Aldrich 451088) and sensor 10 with triethanolamine (Merck 8377). Nitrogen was Alphagaz from ArLíquido.

Compounds used to test the sensors’ sensitivity were: 2,3-butanedione (Aldrich B85307), 2-butanone (Fluka 4380), butyraldehyde (Aldrich W221902), 2-heptanone (Fluka 68592), 2-nonanone (Aldrich 108731), 2-pentanone (Fluka 68950), 2-undecanone (Fluka 68160), 3-(methylthio)-propionaldehyde (Aldrich 277460), acetic acid (Riedel-de-Haën 27264), butyric acid (Riedel-de-Haën 27626), isovaleric acid (Fluka 59850), dimethyl disulfide (Fluka 40221), ethyl hexanoate (Aldrich 148962), and isovaleraldehyde (Aldrich 146455).

Table 1 lists the 57 analysed cheeses. A code composed of two letters and a number is used to identify the cheeses throughout the paper.

### 2.2. Instrumentation

#### 2.2.1. Microextraction of Volatiles

A 75 mm Carboxen-polydimethylsiloxane (CAR-PDMS) SPME fibre (Supelco 57318) was used to extract the volatiles from the headspace of a vial containing each cheese sample.

#### 2.2.2. Electronic Nose

**Figure 1.**
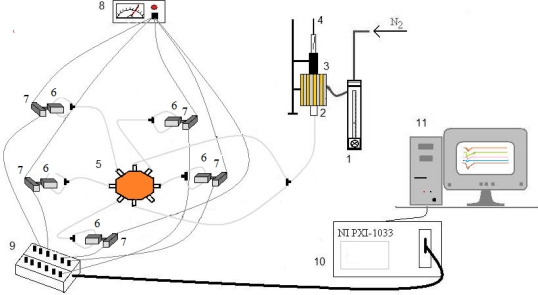
Experimental layout: 1: flowmeter, 2: oven, 3: injection port, 4: SPME fibre, 5: distribution valve, 6: quartz crystal cells, 7: oscillators, 8: power supply, 9: BNC box, 10: Counter/Timer device NI PXI 1033, 11: PC.

**Figure 2.**
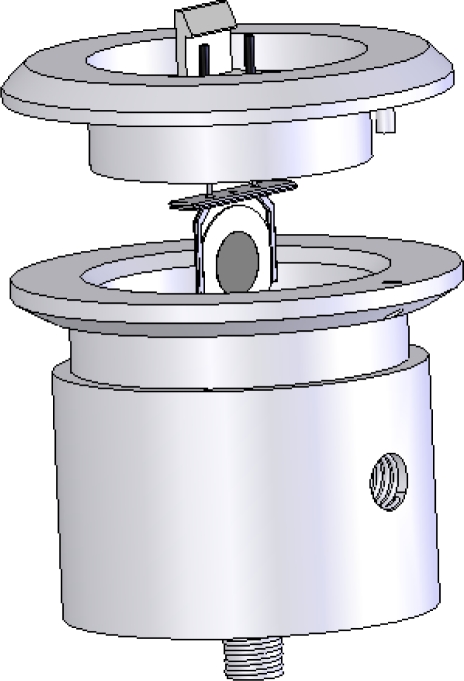
PVC cell housing the coated quartz crystal.

Figure 1 shows the experimental layout. Five sensors were used simultaneously for each experiment. The first experiments were performed with sensors 1 to 5 and then, as data was found to be insufficient to distinguish some cheeses, a new set of experiments was performed using sensors 6 to 10. Each sensor was housed in a separate cell, connected to an oscillator. A distribution valve (OMNIFIT 1103) diverted the gas stream to the sensors. In order to improve contact with the two coated faces of the crystal, the gas flow was further divided in two streams at the entrance to each cell, each one directed to the centre of the coated crystal. Figure 2 shows one of the PVC cells that housed the piezoelectric quartz crystals.

A constant nitrogen flow was maintained through the system. The flow was controlled by a flowmeter (Cole Parmer), placed upstream from the distribution valve. Between the flow controller and the distribution valve, a homemade oven with a septum allowed introduction of the SPME fibre, and the thermal desorption of the cheese sample volatile compounds.

The frequencies of oscillation of the sensors were simultaneously monitored and stored on a PC at intervals of 1 second, using a Counter/Timer device PXI 1033, from National Instruments, and software written in LabView.

### 2.3. Procedure

#### 2.3.1. Sensitivity Evaluation

A flask, with a screw cap with a septum and connected to a smaller flask, was flushed with nitrogen. The compounds under study, usually found in the cheese bouquet, were introduced in both flasks and thermostated in a bath, at 20, or 25 °C, temperatures at which the vapour pressure of the compounds under study were known. An opened tube, immersed into the liquid of the second flask, allowed equilibration at atmospheric pressure, while trapping undesired soluble volatiles. Samples of first flask headspace were withdrawn with a syringe. The exact concentration of the compound in the syringe could be calculated, after which the sample was injected through the injection port of the electronic nose, and the responses of all the five sensors were recorded. By injecting known volumes, a calibration graph could be plotted, and the slope of the linear portion of the calibration curve (sensitivity), could be obtained after least squares fitting.

#### 2.3.2. Cheese Analysis

The SPME fibre was cleaned in a homemade oven, at ∼230 °C, and the complete desorption of compounds was confirmed by analysing the frequency of oscillation of the quartz crystals.

Cheese (2.0 g) was weighed in a 10 mL vial, which was then closed with a silicon septum coated with Teflon, and a removable centre crimp seal. After storage at 4 °C for 24 h, the vials were immersed in a water bath at 30 °C, and a SPME fibre was introduced into the vial headspace for exactly 30 minutes.

Meanwhile, nitrogen was continuously flowing through all the sensors (total flow of 30 mL/mim), and baseline frequencies were recorded. The SPME fibre was then inserted into the oven, and the compounds were desorbed and flushed to the five crystals. The frequencies of the five sensors were simultaneously displayed on the PC monitor, and saved to disc at 1 s intervals. The minimum frequency values were recorded for each sensor, and the difference to the corresponding baseline frequencies computed.

The frequencies of the piezoelectric crystals decreased due to the compounds interacting with the coatings after which they increased again, and reached the baseline values, as soon as desorption from the fibre, as well as from the coatings of the piezoelectric quartz crystals, was completed. The electronic nose and fibre were then ready for a new analysis. The reported frequency shift values for each cheese and each sensor was the mean of four or five replicate analyses.

## 3. Results and Discussion

Table 2 shows the frequency decrease for the piezoelectric quartz crystals due to coating. Assuming that the Sauerbrey equation [16] could be applied for these coatings, *i.e.*, that they were all thin rigid films, which is very unlikely, at least for the polymeric coatings, the amount of coating was estimated to range from 9 to 113 μg. The coatings were selected keeping in mind that they must physically adsorb the volatile compounds reported to be responsible for the cheese bouquets. The interaction between the sensor coatings and the volatile compounds must not involve the establishment of chemical covalent bonds, as this would prevent sensor reversibility. Selectivity is not an issue in electronic noses, but different sensitivities to the target compounds from the different sensors is mandatory.

**Figure 3.**
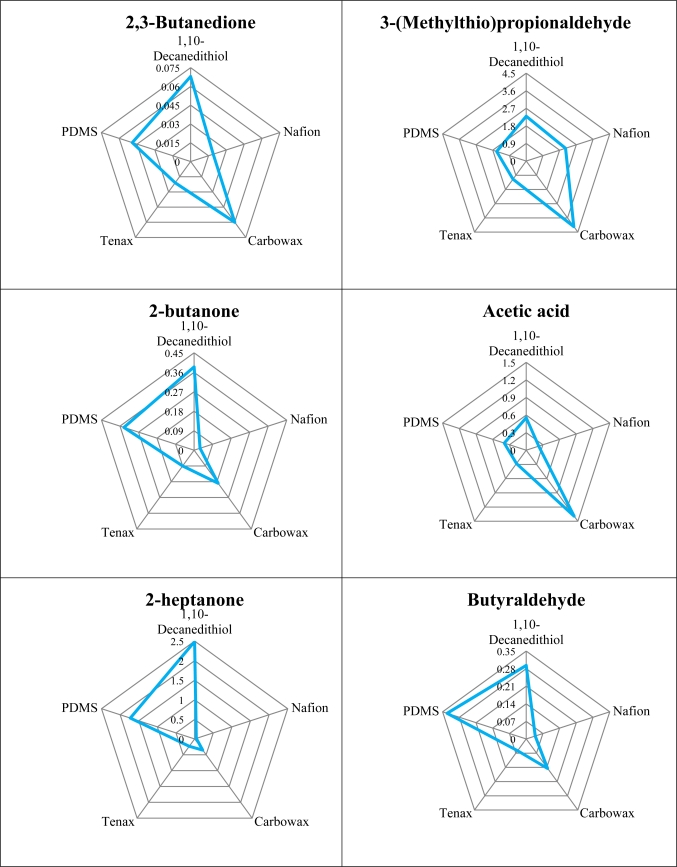

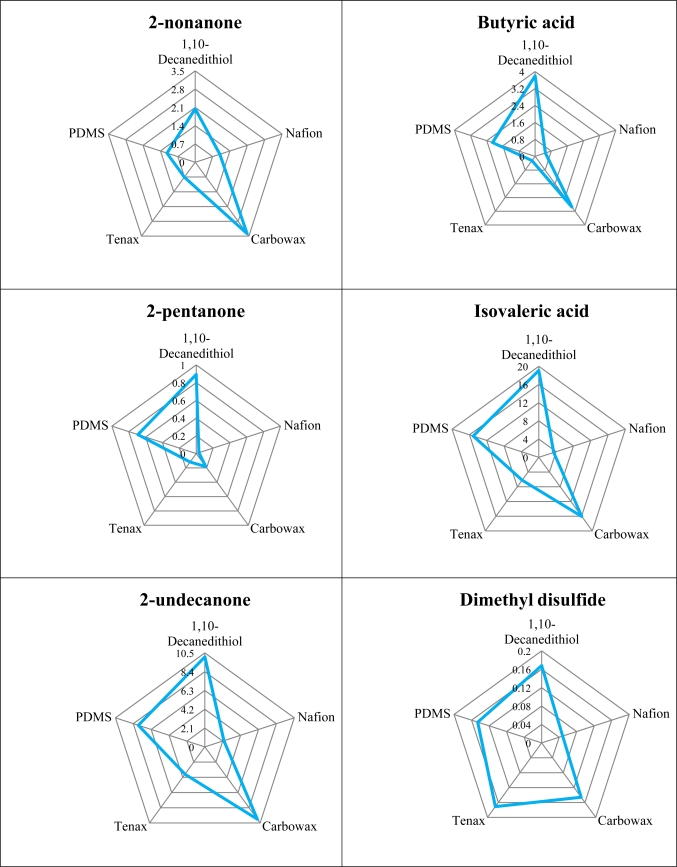

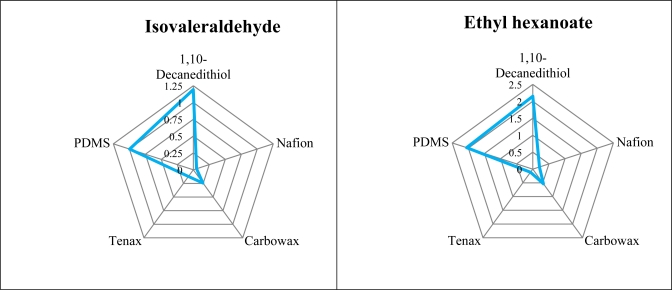
Sensitivities (Hz/μg) of the five sensors to a few volatiles ordinarily present in cheese headspace.

Figure 3 shows the sensitivity of the first five sensors to 14 compounds that, according to the literature, appear in the bouquet of many cheeses. These were selected because they were known to appear in high quantities in some of the selected cheeses, although many other compounds could have been tested, as more than 600 volatile compounds have been identified in cheeses [17]. Sensor 3, coated with Carbowax, was the most sensitive to 3-(methylthio)propioanaldehyde, as well as to acetic acid, 2-nonanone, and 2-undecanone. This last compound was also detected with similar sensitivity using sensor 1, coated with 1,10-decanedithiol. This sensor was also the most sensitive to 2,3-butanedione, 2-butanone. 2-heptanone, butyric acid, 2-pentanone, isovaleric acid, isovaleraldehyde and ethyl hexanoate (sensitivity very similar to sensor 5), although very low sensitivity for the first two compounds was observed. Butyric acid is one of the most important short chain free fatty acids in ewes’ cheese [18], and could also have been detected with high sensitivity by sensor 3 (Carbowax coated). Sensor 5, coated with poly(dimethylsiloxane) – PDMS, was the most sensitive to butyraldehyde, although overall sensitivity to this compound was low. Sensor 4, coated with Tenax, was the most sensitive to dimethyl disulfide.

Sensitivities were not only defined by the coatings applied to the sensors, but also by the ability of the fibre to extract the compounds from the headspace. The SPME fibre used was coated with Carboxen-PDMS and has been shown to be capable of extracting alkanes, esters, methyl ketones, carboxylic acids, as well as sulphur compounds [19].

Its affinity to each individual compound was a major influence on the responses obtained with the sensors and therefore overall sensitivity to the different compounds depended both on the coating sensor and on the fibre.

**Figure 4.**
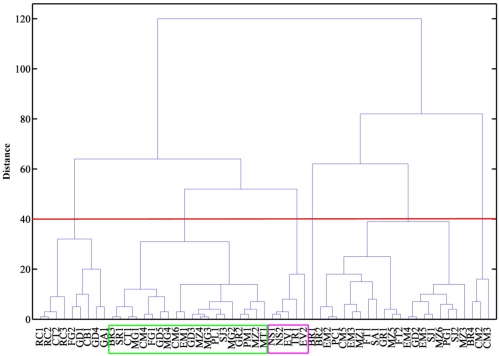
Dendrogram based on Chebyshev distance and complete linkage, applied to data obtained with sensor 3 and sensor 2.

**Figure 5.**
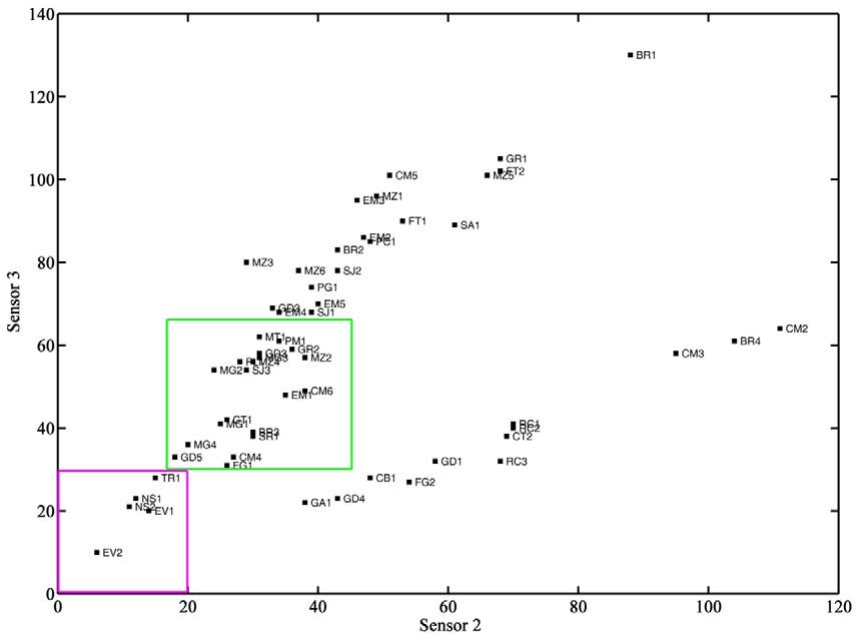
Plot of the responses of sensor 3 *vs.* sensor 2.

Figure 4 shows the dendrogram obtained based on data from sensor 2 and sensor 3, with Chebyshev distances and complete linkage. It can be seen that a first group contained five of the ten ewe cheeses, and that the other five ewe cheeses (four Manchego cheeses and one Seia cheese) belonged to another group, where one cheese made of a mixture of ewes’, goats’ and cows’ milk, goat cheese, buffalo cheese and some cows’ milk cheeses could also be found. Most of the cows’ milk cheeses (32) were excluded from these groups. Figure 5 shows a plot of the frequency decreases observed for sensor 3 *vs.* sensor 2, where the groups containing ewes’ cheese have been marked. It can be concluded that ewe cheeses are characterized by low frequency shift responses on both sensors. Analytical signals of less than 30 on sensor 3 and 20 on sensor 2 could be undoubtedly assigned as belonging to ewes’ cheese. Cheeses giving rise to signals between 30 and 60 on sensor 3 and between 18 and 45 on sensor 2 could also be from ewes’ cheese, but classification was not solid. Outside these limits, no cheese made from ewes’ milk could be found.

The distinction between ewe cheeses and cheeses made from cows’ milk, and especially from cheeses made from a mixture of milks is very important from a commercial point of view, as the addition of cows’ milk to ewes’ milk is common. This work allowed us to identify half of the ewe cheeses, but left the other half in a grey area, where the origin could not be confirmed. All cheeses outside these two groups could however be confirmed as non-ewes’ cheese. We are only aware of one other study [14], based on an electronic nose composed of chemical sensors, where cheeses made from goats’ milk, were effectively separated from cows’ milk cheeses. Although in this study all the milk was from the same origin. In the present study, cheese samples were commercial, from a variety of different sources, and the milk came from many different sources. Cheese manufacturing conditions were also different, and ripening stage was unknown. No other information on the cheeses besides that displayed on the label was available. A recent work [13] where ewes’ milk cheeses have been analyzed by an electronic nose and GC-MS, showed that significant differences in the bouquet arose due to ripening time and manufacturing technique. Climate, geology, forage and breed also influence milk quality [20], and it is expected therefore, that the problem of detecting fraud in ewes’ cheese to be less demanding than the scenario presented here, as information on cheese variety, origin and ripening time would be available to inspectors.

The five sensors used initially to distinguish cheeses made from ewes’ milk from cheeses made from other milks did not allow the cheese variety to be distinguished. Therefore, 31 new samples of some of the varieties previous analyzed were in contact with five new sensors. The sensitivity of the new sensors to some of the compounds commonly found in cheeses was not studied, but they were chosen based on previous knowledge that existed in our laboratory. For instance, Mn-pht coating was known to respond to alkanes [21], and TEA to sulfur compounds [22].

**Figure 6.**
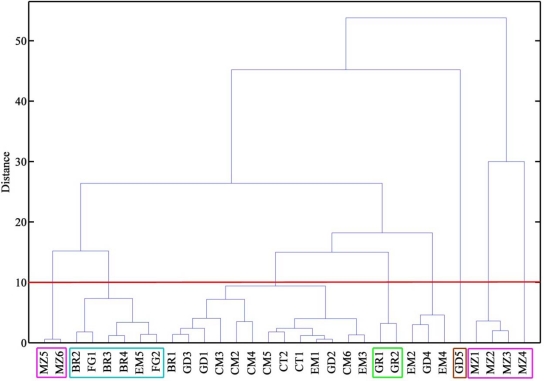
Dendrogram based on Chebyshev distance and complete linkage, applied to data obtained with sensor 10 and sensor 8.

**Figure 7.**
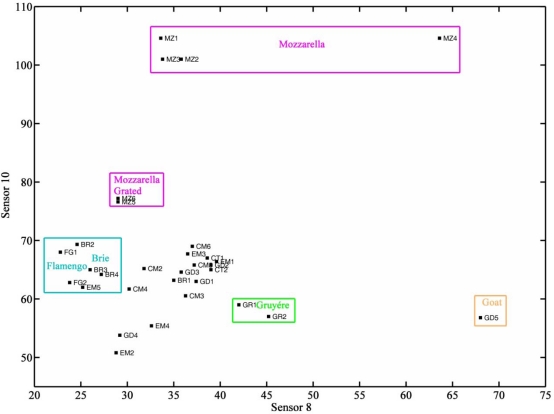
Plot of the responses of sensor 10 *vs.* sensor 8.

Figure 6 shows the result of a cluster analysis made with data obtained with sensor 10 and sensor 8, also based on Chebyshev distance and complete linkage. Brie and Flamengo cheeses, except for Brie coeur de lion, which had a different origin, were clearly separated. Mozzarella cheeses belonged to a single group, with Mozzarella grated cheeses also separated. Gruyère cheeses were also distinguished. There was only one goats’ cheese (a Gouda cheese) which was also distinguished by sensors 8 and 10. Figure 7 shows a plot of data obtained with sensor 10 *vs.* data obtained with sensor 8. Groups have been marked on the plot. Mozzarella cheeses were characterized by very high responses from sensor 10. Among cheeses with moderate responses from sensor 10, Brie and Flamengo cheeses were characterized by low responses on sensor 8 (less than 30), Gruyère cheeses by responses between 40 and 50, and the goats’ cheese by very high signals (>65).

## 4. Conclusions

Two sensors coated with Nafion (sensor 2) and Carbowax (sensor 3) were able, in the absence of any other information, to certify that labelled ewe cheeses were effectively produced from ewes’ milk (responses of less than 30 on sensor 3 and 20 on sensor2), to assign them for possible certification after further analysis by other methods (responses between 30 and 60 on sensor 3 and between 18 and 45 on sensor 2) or to exclude this hypothesis. Two sensors, sensor 8 and sensor 10, coated with polyvinylpyrrolidone and triethanolamine, respectively, were able to distinguish between Mozzarella cheeses (responses higher than 75 on sensor 10), Flamengo and Brie (responses lower than 27 on sensor 8 and between 62 and 70 on sensor 10), and Gruyère (bellow 75 on sensor 10, and between 42 and 46 on sensor 8). These four sensors will be used, in the future, to detect frauds and to confirm authenticity of cheeses with a certificate of origin.

## Figures and Tables

**Table 1.** Analysed cheeses.

**Code**	**Cheese**	**Origin**	**Milk**

BR1	Brie Coeur de lion	FR 50.168.01 CE	Cow
BR2	Brie Etoile d’or	FR 88.115.01 CE	Cow
BR3	Brie Pointe de Bridel	FR 88.115.01 CE	Cow
BR4	Brie Président	FR 88.115.01 CE	Cow
CB1	Cabra Transmontano	PT TLT1054 CE	Goat
CM2	Camembert Cantorel	FR 15.196.01 CE	Cow
CM3	Camembert Crémiere de France	FR 56.179.01 CE	Cow
CM4	Camembert Emile Bridel	FR 50.453.01 CE	Cow
CM5	Camembert Le chêne d'argent	FR 61.145.01 CE	Cow
CM6	Camembert Presidént	FR 50.453.01 CE	Cow
CT1	Comté EntreMont	FR 36.558.01 CE	Cow
CT2	Comté seléction t	FR 25.601.003 CE	Cow
EM1	Emmental Coeur de Meule	FR 53.061.01 CE	Cow
EM2	Emmental EntreMont	FR 74.010.61 CE	Cow
EM3	Emmental Milbona	DE-BY-301 EG	Cow
EM4	Processes Emmental Tenery	DE-BW-470 EG	Cow
EM5	Emmental Rapê Etoile d’or grated	FR 44.023.001 CE	Cow
EV1	Évora	PT TLT740 CE	Ewe
EV2	Évora	PT TLT444 CE	Ewe
FG1	Flamengo Terra Nostra	PT DLT110 CE	Cow
FG2	Flamengo Agros	PT BLT7CE	Cow
FT1	Feta Dionis	EL 35.3.1026 EEC	Goat and Ewe
FT2	Feta Grego	GR 20.2.200 EU	Cow and Goat
GD1	Gouda Jung & Mild Benjamim	DE-NI-086 EG	Cow
GD2	Processed Gouda Milbona	DE-NI-058 EG	Cow
GD3	Processed Gouda Tenery	DE-BW-470 EG	Cow
GD4	Gouda Westland	NL	Cow
GD5	Gouda Zikko Westland	NL	Goat
GA1	Grana Padano	IT 03/267 CE	Cow
GR1	Gruyere EntreMont	FR 39.558.01 CE	Cow
GR2	Gruyere Emmi	CH 2038	Cow
MG1	Manchego	ES 15.00751/CR	Ewe
MG2	Manchego El Mesonero	ES 15.047955/AB	Ewe
MG3	Manchego Flor de Mi Pueblo	ES 15.03173/V	Ewe
MG4	Manchego Garcia Baquero	ES 15.00229/CR	Ewe
MT1	Fresh Matinal	ES 15.00905/0 CE	Cow
MZ1	Mozzarella Lovilio	DE-BY-301 EG	Cow
MZ2	Mozzarella Granarolo	IT 09.10 CE	Cow
MZ3	Mozzarella Solo Italia	IT 41-5 CE	Cow
MZ4	Mozzarella Negrino	IT 15/332 CE	Buffalo
MZ5	Grated Mozzarella Ramazzotti	DK-M206 EC	Cow
MZ6	Grated Mozzarella Lovilio	DE-BY-301 EG	Cow
NS1	Nisa Qual	PT LLT1463 CE	Ewe
NS2	NisaMonforqueijo	PT LLT-663 CE	Ewe
PC1	Fresh Paiva	PT TLT36 CE	Goat
PL1	Fresco Paiva light	PT TLT36 CE	Cow
PG1	Fresh Paiva semi skimmed	PT TLT36 CE	Cow
PM1	Fresh Paiva mixture	PT TLT36 CE	Cow, Goat and Ewe
RC1	Raclette Classique Emmi	CH 2066	Cow
RC2	Raclette Président	FR 25.601.03 CE	Cow
RC3	Raclette Saveur D’Antan	FR 22.061.15 CE	Cow
SA1	St Albray	FR	Cow
SJ1	São Jorge 3 months	PT ALT516 CE	Cow
SJ2	São Jorge 4 months	PT ALT516 CE	Cow
SJ3	São Jorge 7 months	PT ALT516 CE	Cow
SR1	Seia	PT-ILT75 CE	Ewe
TR1	Terrincho	PT TLT668 CE	Ewe

**Table 2.** sensors’ coatings, and frequency decreases due to coating.

**Sensor**	**Coating**	**Frequency decrease due to coating (kHz)**

1	1,10-decanedithiol	4.2
2	Nafion	34.7
3	Carbowax	51.1
4	Tenax	23.8
5	poly(dimethylsiloxane)	23.3
6	manganese(II) phthalocyanine	25.8
7	poly(vinyl alcohol)	21.1
8	polyvinylpyrrolidone	17.2
9	6-mercapto-1-hexanol	7.6
10	triethanolamine	12.6

## References

[1] Ampuero S., Bosset J.O. (2003). The electronic nose applied to dairy products: A review. Sens. Actuat. B.

[2] Pillonel L., Ampuero S., Tabacchi R., Bosset J.O. (2003). Analytical methods for the determination of the geographic origin of Emmental cheese: Volatile compounds by GC/MS-FID and electronic nose. Eur. Food Res. Technol.

[3] Pérès C., Begnaud F., Eveleigh L., Berdagué J.L. (2003). Fast characterization of foodstuff by headspace mass mass spectrometry (HS-MS). Trends Anal. Chem.

[4] Drake M.A., Gerard P.D., Kleinhenz J.P., Harper W.J. (2003). Application of an electronic nose to correlate with descriptive sensory analysis of aged Cheddar cheese. LTW Food Sci. Technol.

[5] Pérès C., Begnaud F., Denoyer C., Berdagué J.L. (2002). Fast characterization of Camembert cheeses by static headspace—Mass spectrometry. Sens. Actuat. B.

[6] Pérès C., Denoyer C., Tournayre P., Berdagué J.L. (2002). Fast characterization of cheeses by dynamic headspace—Mass spectrometry. Anal. Chem.

[7] Pozo-Bayón M.-A., Martín-Álvarez P.J., Reineccius G.A. (2009). Monitoring changes in the volatile profile of cheese crackers during storage using GC-MS and PTR-MS. Flavour Frag. J.

[8] Wu Z., Chingin K., Chen H., Zhu L., Jia B., Zenobi R. (2010). Sampling analytes from cheese products for fast detection using neutral desorption extractive electrospray ionization mass spectrometry. Anal. Bioanal. Chem.

[9] Fernandes D.L.A., Gomes M.T.S.R. (2008). Development of an electronic nose to identify and quantify volatile hazardous compounds. Talanta.

[10] Trihaas J., Vognsen L., Nielsen P.V. (2005). Electronic nose: New tool in modelling the ripening of Danish blue cheese. Int. Dairy J.

[11] O’Riordan P.J., Delahunty C.M. (2003). Characterisation of commercial Cheddar cheese flavour. 1: Traditional and electronic nose approach to quality assessment and market classification. Int. Dairy J.

[12] O’Riordan P.J., Delahunty C.M. (2003). Characterisation of commercial Cheddar cheese flavour. 2: Study of Cheddar cheese discrimination by composition, volatile compounds and descriptive flavour assessment. Int. Dairy J.

[13] Cevoli C., Cerretani L., Gori A., Caboni M.F., Toschi T.G., Fabbri A. (2011). Classification of Pecorino cheeses using electronic nose combined with artificial neural network and comparison with GC-MS analysis of volatile compounds. Food Chem.

[14] Haddi Z., Annanouch F., Amari A., Hadoune A., Bouchikhi B. Application of a Portable Electronic Nose Device to Discriminate and Identify Cheeses with Known Percentages of Cow’s and Goat’s Milk.

[15] Bargon J., Braschoβ S., Flörke J., Herrmann U., Klein L., Loergen J.W., Lopez M., Maric S., Parham A.H., Piacenza P. (2003). Determination of the ripening state of Emmental cheese via quartz crystal microbalances. Sens. Actuat. B.

[16] Sauerbrey G. (1959). Verwendung von Schwingquartzen zur Wägung dünner Schichten und zur Mikrowägung. Z. Phys.

[17] Curioni P.M.G., Bosset J.O. (2002). Key odorants in various cheese types as determined by gas chromatography-olfactometry. Int. Dairy J.

[18] Pinho O., Ferreira I.M.P.L.V., Ferreira M.A. (2002). Solid-phase microextraction in combination with GC/MS for quantification of the major volatile free fatty acids in ewe cheese. Anal. Chem.

[19] Pérès C., Viallon C., Berdagué J.L. (2001). Soli-phase microextraction—Mass spectrometry: A new approach to the rapid characterization of cheeses. Anal. Chem.

[20] Pillonel L., Badertscher R., Bütikofer U., Casey M., Dalla Torre M., Lavanchy P., Meyer J., Tabacchi R., Bosset J.O. (2002). Analytical methods for the determination of the geographic origin of Emmentaler cheese. Main framework of the project; chemical, biochemical, microbiological, colour and sensory analyses. Eur. Food Res. Technol.

[21] Fernandes D.L.A., Oliveira J.A.B.P., Gomes M.T.S.R. (2008). Detecting spoiled fruit in the house of the future. Anal. Chim. Acta.

[22] Fernandes D.L.A., Rolo T.A., Oliveira J.A.B.P., Gomes M.T.S.R. (2009). A new analytical system, based on an acoustic wave sensor, for halitosis evaluation. Sens. Actuat. B.

